# Epidemiology and antifungal susceptibilities of rare yeast infections in a tertiary care center

**DOI:** 10.1186/s12866-025-04016-1

**Published:** 2025-07-02

**Authors:** Berire Yavuz, Özge Turhan, Özlem Koyuncu Özyurt, Özgül Çetinkaya, Çağlayan Merve Ayaz, Betil Özhak, Latife Mamıkoğlu

**Affiliations:** 1 Department of Infectious Disease and Clinical Microbiology, Faculty of Medicine, Akdeniz University, Antalya, Turkey; 2 Department of Medical Microbiology, Faculty of Medicine, Akdeniz University, Antalya, Turkey; 3 Department of Medical Microbiology, Ministry of Health Antalya Training and Research Hospital, Antalya, Turkey

**Keywords:** Antifungal susceptibility, Fungemia, Candidemia, Yeasts

## Abstract

**Background:**

With advancements in healthcare services, there has been an increase in the population of immunosuppressed patients and the frequency of opportunistic pathogens including rare yeasts (RY). We aimed to provide data on the distribution of RY causing invasive infections and their antifungal susceptibility profiles. In this retrospective study, the distribution of RYs obtained between January 2015 and January 2023 examined. Antifungal susceptibility analyses of the isolates conducted using the broth microdilution method following European Committee on Antimicrobial Susceptibility Testing (EUCAST) recommendations. The patients’ demographics, predisposing factors and outcomes were investigated.

**Results:**

A total of 15 species were identified from 56 clinical samples, including *Clavispora lusitaniae*, *Kluyveromyces marxianus*, *Magnusiomyces capitatus*, *Wickerhamomyces anomalus*, *Meyerozyma guilliermondii*, *Magnusiomyces clavatus*, *Candida dubliniensis*, *Rhodotorula mucilaginosa*, *Cyberlindnera jadinii*, *Pichia inconspicua*, *Candida auris*, *Yarrowia lipolytica*, *Trichosporon asahii*, *Wickerhamiella pararugosa* and *Saccharomyces cerevisiae*. Observed highly heterogenous antifungal susceptibility results depending on the species. Admission to the intensive care unit (ICU), being 65 years or older, and neutropenia increased mortality. Fourteen-day and 30-day mortality rates were 27.8% and 35.2%, respectively.

**Conclusions:**

The variability of RY encountered in various centers and differences in antifungal susceptibility profiles warrant further research on this topic, both regionally and globally.

**Supplementary Information:**

The online version contains supplementary material available at 10.1186/s12866-025-04016-1.

## Background

Invasive fungal infections (IFIs) are predominantly encountered in immunosuppressed and critically ill patients. With advancements in treatment options, there has been an increase in the number of immunosuppressed patients. These patients are more prone to infections, including opportunistic pathogens [1]. Yeasts constitute a portion of these pathogens and are considered a significant cause of morbidity and mortality [2, 3]. Rare yeast infections are reported as outbreaks or sporadic cases in hemato-oncology patient care centers and ICUs [4–7]. Fungal infections can manifest in various clinical conditions ranging from superficial and mucosal involvement to invasive diseases. Early diagnosis, initiation of appropriate antifungal therapy and source control are crucial in patients with IFIs [8]. The main cause of IFIs is typically *Candida albicans*, accounting for about 65–70% of cases. However, there has been an increase in the prevalence of non-albicans *Candida* species and other RYs in recent years especially in immunosuppressed patients [9–11]. Examples of RY include *Clavispora lusitaniae* (formerly *Candida lusitaniae*), *K. marxianus* (formerly *Candida kefyr*), *W. anomalus* (formerly *Candida pelliculosa*, *Pichia anomala*), *M. capitatus* (formerly *Saprochaete capitata*, *Geotricum capitatum*, *Blastoschizomyces capitatus*), *M. clavatus* (formerly *Saprochaete clavate*, *Geotrichum clavatum*), *C. jadinii* (formerly *Pichia jadinii*, *Candida utilis*), *P. inconspicua* (formerly *Candida inconspicua*), *M. guilliermondii* (formerly *Candida guilliermondii)*, *Y. lipolytica* (formerly *Candida lipolytica*), and *W. pararugosa* (formerly *Candida pararugosa*). Antifungal susceptibility analyses have shown varying degrees of resistance to various antifungal agents in these pathogens as well [3, 12–14]. *C. auris* is a highly virulent species that was first identified in Japan in 2009. This species is frequently resistant to numerous antifungal agents and has been known to cause outbreaks with high mortality rates. The pathogen exhibits a strong ability to colonize the skin, mucous membranes, and environmental surfaces within healthcare settings, with its survival on dry surfaces outside the host playing a key role in hospital-associated outbreaks [15, 16].

Unfortunately, data on the susceptibility profiles of RYs remain limited in the literature. The aim of this study was to provide data on the distribution of RYs causing invasive infections and their antifungal susceptibility profiles.

## Methods

### Study design

Rare yeasts obtained from clinical samples received at the Central Laboratory of the Department of Medical Microbiology, Akdeniz University Faculty of Medicine, between January 2015 and January 2023, were included. The clinical findings of the patients were retrospectively reviewed. Patients were divided into three categories according to age: under 18 years, 18–64 years and 65 years and over. The analysis covered cultures from samples (blood, catheter, pleural, peritoneal, synovial fluid) from a total of 92 patients. In cases where multiple isolates of the same pathogen were found in a single patient, only the first identified pathogen was included in the study. Infections with different pathogens at different times in two patients with prolonged hospitalization (> 6 months) were evaluated separately.

### Identification methods and susceptibility testing

Identification of the isolates were performed by conventional methods (e.g., macroscopic examination of cultures, microscopic examination of growth, germ tube test, morphology on corn meal agar with tween 80, urease test) and Bruker Biotyper v3.1 (Bruker Daltonics, Germany) using the ethanol-formic acid extraction method according to the manufacturer’s recommendations. The identification data generated by MALDI-TOF MS were classified following the manufacturer’s instructions: a log score value ≥ 2.0 indicated species identification, a score of 1.7–1.99 indicated identification at the genus level, and a score < 1.7 indicated non-reliable identification. Invitro antifungal susceptibility analysis was performed by microdilution method according to the recommendations of EUCAST [17]. Microdilution plates were read with a microdilution plate reader (Bio-Rad). A wavelength of 405 nm was used to measure the absorbance value. For amphotericin B, the lowest concentration at which the absorbance value measured in the growth control decreased by ≥ 90% was taken as the MIC value, while for other antifungals, the lowest drug concentration at which the absorbance value decreased by ≥ 50% was accepted as the MIC value. Isolates that did not show viability and isolates that did not achieve sufficient absorbance values in the reading of the microdilution plates were excluded from the study. As a result of all these procedures, a total of 57 isolates, including 56 yeasts from 54 patients and *C. parapsilosis* ATCC 22019 control strain, were included in the study.

Interpretation of MIC values for RYs was based on EUCAST clinical breakpoints (version 11.0) when available. However, for the most RY species, there were no established breakpoints. For isolates without defined breakpoints, interpretations were made based on ECOFF values (version 5.0) determined by EUCAST or compared with the results of relevant published studies where antifungal susceptibility testing was performed using the EUCAST methodology.

### Definitions

Patients with a total neutrophil count below 500 cells/mm^3^ were considered neutropenic. Peripherally inserted catheters and port catheters were considered central venous catheters. Patients who had been receiving antibiotics for at least three days at the time of fungemia were considered as patients with prior antibiotic exposure. In the surgical history, major abdominal, thoracic, and cranial operations performed up to 30 days prior to the IFIs were evaluated.

### Statistical analysis

All categorical data are presented as numbers (percentages) and all numerical data as medians (interquartile range, IQR). Where applicable, the Mann-Whitney U test was used to compare continuous numerical variables that were not normally distributed, whereas the Pearson’s χ^2^ test or Fisher’s exact test was used to compare categorical variables. Multivariate logistic regression analysis was conducted to evaluate the hazard ratio (HR) with 95% confidence intervals (CI) for the effect of demographic, clinical and laboratory predictors on 14-day and 30-day mortality. A *p*-value of less than 0.05 was considered statistically significant. The statistical package SPSS for Windows, version 23.0 (IBM SPSS Statistics, IBM Corporation, Armonk, NY, United States) was used for the analysis.

## Results

Fifty-six RY were obtained from a total of 54 patients. The hospitalization of two pediatric patients was over six months and two different yeasts isolated in two different blood cultures taken at different times were evaluated separately. A total of 15 species were identified over an eight-year period from 2015 to 2023. The most frequently isolated species were *C. lusitaniae* (*n* = 14), followed by *K. marxianus* (*n* = 12), *M. capitatus* (*n* = 5), *W. anomalus* (*n* = 4), *M. guilliermondii* (*n* = 4), *M. clavatus* (*n* = 3), *C. dubliniensis* (*n* = 3), *R. mucilaginosa* (*n* = 2), *C. jadinii* (*n* = 2), *P. inconspicua* (*n* = 2), *C. auris* (*n* = 1), *Y. lipolytica* (*n* = 1), *T. asahii* (*n* = 1), *W. pararugosa* (*n* = 1), and *S. cerevisiae* (*n* = 1). One isolate was obtained from a pleural culture, while the others were detected in the blood samples. There was diversity in antifungal susceptibility analysis among the species. MIC value ranges of the isolates are shown in Table 1.

**Table 1:** Distribution of fungal isolates and minimum inhibitory concentration values ranges

Species (*n*)	MIC value (mg/L)							
	AMB	FLC	VRC	POS	ISA	AFG	MFG	CAS
*Clavispora lusitaniae* (**14**)	0.031-0.5	< 0.125-1	< 0.008–0.015	< 0.008–0.125	< 0.008	0.031–0.25	< 0.008–0.125	0.125-0.5
*Kluyveromyces marxianus* (**12**)	0.0625-0.5	0.25-32	< 0.008-2	< 0.008-0.5	< 0.008–0.125	< 0.008–0.25	< 0.008–0.125	0.031-0.5
*Magnusiomyces capitatus* (**5**)	0.5-1	32->64	2- >4	4- >4	2- >4	> 4	> 4	> 4
*Wickerhamomyces anomalus* (**4**)	0.015–0.25	4	0.125–0.25	0.0625-2	< 0.008–0.031	0.008–0.0625	< 0.008–0.008	0.125–0.25
*Meyerozyma guilliermondii* (**4**)	0.0625-0.5	4–8	0.25–0.5	0.25–0.5	0.125-0.5	0.125-4	0.125-0.5	0.5-1
*Magnusiomyces clavatus* (**3**)	0.25	2->64	0.015->4	1->4	0.008- >4	> 4	0.25->4	> 4
*Candida dubliniensis* (**3**)	0.125–0.25	0.25–0.5	< 0.008–0.008	0.015–0.031	< 0.008	0.008-4	0.008-1	0.0625-1
*Pichia inconspicua* (**2**)	0.125–0.25	32->64	0.5->4	0.25-1	0.125–0.25	0.125–0.25	0.031	0.015-0.5
*Cyberlindnera jadinii* (**2**)	0.0625–0.125	1–2	0.0625–0.125	< 0.008–0.25	0.008	0.015	< 0.008	0.031-1
*Rhodotorula mucilaginosa* (**2**)	0.5-1	64->64	1->4	0.5- >4	1- >4	> 4	> 4	> 4
*Saccharomyces cerevisiae* (**1**)	1	> 64	> 4	> 4	> 4	> 4	0.0625	1
*Yarrowia lipolytica* (**1**)	0.5	8	> 4	> 4	> 4	0.5	1	1
*Candida auris* (**1**)	0.5	64	0.25	0.125	< 0.008	0.5	0.0625	0.125
*Trichosporon asahii* (**1**)	0.031	2	0.031	0.5	0.008	> 4	> 4	> 4
*Wickerhamiella pararugosa* (**1**)	0.125	1	0.25	0.25	0.25	1	2	0.5

Abbreviations: AFG, anidulafungin; AMB, amphotericin B; CAS, caspofungin; FLC, fluconazole; ISA, isavuconazole; MFG, micafungin; MIC, minimal inhibitory concentration; POS, posaconazole; VRC, voriconazole

*Clavispora lusitaniae* was the most encountered RY species followed by *K. marxianus* and *M. capitatus*. According to EUCAST clinical breakpoints for *C. dubliniensis*, all isolates were susceptible to amphotericin B, voriconazole, fluconazole, and posaconazole; however, one isolate exhibited resistance to both anidulafungin and micafungin. With the exception of *C. dubliniensis*, no other breakpoints have been established by EUCAST for the RYs in the present study. However, ECOFF values are available for some RYs. While ECOFFs do not serve as clinical breakpoints and cannot predict therapeutic success alone, they are necessary to distinguish between wild-type and non-wild-type populations, which is critical for monitoring emerging resistance. All *C. lusitaniae* isolates exhibited amphotericin B and micafungin MIC values at or below the ECOFF value, whereas anidulafungin MIC values in four isolates were found to be above the ECOFF value for this species. For *K. marxianus*, the fluconazole MICs of four isolates were above the ECOFF value, while anidulafungin MICs were higher than the ECOFF value in five isolates. In contrast, all *K. marxianus* isolates had amphotericin B and micafungin MICs at or below the ECOFF values. All *M. guilliermondii* isolates exhibited MIC values at or below for fluconazole, micafungin and amphotericin B, whereas one isolate showed an anidulafungin MIC value higher than the ECOFF value. Additionally, all *M. guilliermondii* isolates exhibited posaconazole MICs that exceeded the established ECOFF for this species. For *M. clavatus*, one strain, only micafungin MIC value was 0.25 mg/L among the echinocandins. For the other strains, high MIC values (> 4 mg/L) were detected for all echinocandins. The MIC values of amphotericin B and anidulafungin for *S. cerevisiae* were found to exceed the ECOFFs, whereas the MIC of micafungin remained below the ECOFF. *C. auris* MIC values were found to be lower than the amphotericin B and micafungin ECOFF values but higher than the anidulafungin ECOFFs for this species. According to the CDC tentative MIC breakpoints for *C. auris* indicate that the isolate demonstrated resistance to fluconazole and sensitivity to amphotericin B, caspofungin, anidulafungin and micafungin.

Thirty-six (66.7%) of the patients were over 18 years. The median age was 42.83 years (IQR 8.63–64.5 years) and 40 patients (74.1%) were male. Fifteen patients (27.8%) had hematologic malignancy and 13 patients (24.1%) had solid organ malignancy. The median duration between hospitalization and isolation was 21.00 days (IQR 10.00–35.00 days). Nineteen patients (35.2%) were diagnosed with sepsis, and 28 patients (51.9%) received total parenteral nutrition (TPN). Of the total number of patients, 26 (48.1%) were monitored in the ICU for a median duration of 8.00 days (IQR 4.75–28.25). Twenty-four patients (44.0%) were receiving corticosteroids. Fifteen out of 54 patients died within the first 14 days (27.8%). 30-day mortality rate was 35.2%. The demographic and clinical characteristics of the patients are summarized in Table 2. Supplementary Table 1 provides further details on the clinical profiles, including concomitant comorbidities, antifungal treatments, clinical outcomes, and antifungal susceptibility results of the isolated agents.

**Table 2:** Demographic and clinical characteristics of patients

Patients (*n*, %)		
Pediatrics	18	33.3
Adults	36	66.7
Age, years, median (IQR)*	42.83 (8.63–64.5)	
Gender (*n*, %)*		
Male	40	74.1
Underlying diseases (*n*, %)	51	94.4
Hematological malignancies	15	27.8
Solid organ tumors	13	24.1
Solid organ transplantations	6	11.1
Hypertension	6	11.1
Hemodialysis	5	9.3
Chronic heart diseases	3	5.6
Cerebrovascular events	3	5.6
Diabetes mellitus	3	5.6
Predisposing factors (*n*, %)		
Use of central venous catheter	43	79,6
History of surgery	17	31.5
Total parenteral nutrition	28	51.9
Neutropenia*	20	37.0
Prior antimicrobial exposure	53	98.1
ICU admission*	26	48.1
ICU, day, median (IQR)	8.00 (4.75–28.25)	
Receipt of corticosteroid	24	44.0
Sepsis	19	35.2
Antifungal prophylaxis (*n*, %)	13	24.1
Between hospitalization and isolation time, day, median (IQR)	21.00 (10.00-35.50)	
Death (*n*, %)	37	68.5
14-day mortality	15	27.8
30-day mortality	19	35.2

Abbreviations: IQR, interquartile range; ICU, intensive care unit

* These variables were evaluated by logistic regression analysis with 14- and 30-day mortality

ICU admission and sepsis were significantly associated with higher 14-day (*p* = 0.004 and *p* < 0.001, respectively) and 30-day (*p* = 0.001 and *p* < 0.001, respectively) mortality rates. A statistically significant association was observed between neutropenia and 14-day mortality (*p* = 0.03). Although TPN administration was higher for both 14-day and 30-day mortality rates, the difference did not reach statistical significance (*p* = 0.10 and *p* = 0.16, respectively).

Multivariate analyses revealed a statistically significant association between 14-day mortality and age 65 years or older (HR, 19.961, [95% CI], 2.037-195.605, *p* = 0.01), neutropenia (HR, 31.081, [95% CI, 2.783-347.084], *p* = 0.005) and ICU stay (HR, 40.121, [95% CI, 3.532-455.762], *p* = 0.003). Thirty-day mortality was significantly associated with age 65 years or older (HR, 14.964, [95% CI, 1.913-117.067], *p* = 0.01), neutropenia (HR, 7.735, [95% CI, 1.246–47.998], *p* = 0.03) and ICU admission (HR, 23.230, [95% CI, 3.613-149.359], *p* = 0.001). The results are given in Table 3.

**Table 3:** Factors associated with all cause 14- and 30-day mortality by logistic regression analysis

	HR	CI 95%	*p* value
14-day mortality			
≥65 of age	19.961	2.037-195.605	0.01
ICU admission	40.121	3.532-455.762	0.003
Neutropenia	31.081	2.783-347.084	0.005
30-day mortality			
≥65 of age	14.964	1.913-117.067	0.01
ICU admission	23.230	3.613-149.359	0.001
Neutropenia	7.735	1.246–47.998	0.03

Abbreviations: HR, hazard ratio; CI, confidence interval; ICU, intensive care unit

In regression analysis with 14-day mortality, age, gender, ICU admission and neutropenia were included (Hosmer and Lemeshow test sig. 0.76). For 30-day mortality, age, gender, neutropenia and ICU admission were included (Hosmer and Lemeshow test sig. 0.50)

## Discussion

Rare yeast infections are characterised by a rapid progression and high mortality, particularly in immunosuppressed patients [1]. This study presents data on the species identified in our center, providing guidance for the early initiation of appropriate antifungal therapy. *Clavispora lusitaniae* was the most encountered RY species in the study. In a study conducted by Astvad et al., a classification of the MIC values for amphotericin B, anidulafungin, fluconazole and voriconazole for RYs is presented, for which no breakpoint has been established by the EUCAST. Based on this classification, the majority of the isolates can be considered to be susceptible to both fluconazole, voriconazole and anidulafungin. In the present study, all isolates exhibited amphotericin B MIC values of ≤ 1 mg/L. However, as noted by Astvad et al. the use of amphotericin B is generally not recommended for this species [18]. In addition, other in vitro antifungal susceptibility analyses suggest that *C. lusitaniae* appears to be susceptible to amphotericin B, although resistance can emerge during treatment [5, 19]. Studies have observed phenotypic resistance to amphotericin B in *C. lusitaniae* strains only when gradient diffusion strips were used and not detected by microdilution methods [20]. In a study by Pfaller et al., 67 *C. lusitaniae* isolates obtained from blood were tested for amphotericin B resistance using the gradient diffusion method and 98% of the strains interpreted as susceptible. In the study, *C. lusitaniae* isolates were considered susceptible to fluconazole, voriconazole and posaconazole [21]. These findings are similar to the susceptibility results of the isolates in our study. As in other studies, the study by Diekema et al. reported that the isolates appeared susceptible to amphotericin B; however, caution in treatment was advised due to the risk of secondary resistance development. The isolates were reported to be quite susceptible to triazoles and echinocandins [22].

*Kluyveromyces marxianus* is commonly found in dairy products and causes nosocomial infections particularly in patients with hematologic malignancies [4]. In the study by Diekema et al., isolates were found to be susceptible to azoles and echinocandins, while high MIC values ​​for amphotericin B were reported [22]. However, the findings of the present study indicated that all isolates were classified as amphotericin B sensitive, eight isolates were classified as fluconazole sensitive, six isolates were classified as voriconazole sensitive and seven were classified as anidulafungin sensitive, as per the classification of Astvad et al. [18]. In the study by Pfaller et al., 10 isolates demonstrated reduced susceptibility to amphotericin B, while remaining susceptible to fluconazole, posaconazole, and voriconazole [21]. In a case reported by Spiliopoulou et al., pyelonephritis caused by *K. marxianus* in a COVID-19 patient followed in the intensive care unit was successfully treated with fluconazole [23].

*Magnusiomyces capitatus* is known to cause systemic infections with a high mortality rate, particularly in immunosuppressed patients [24, 25]. Case reports and various outbreaks have been reported in the literature and treatment with amphoterisin B, voriconazole or posaconazole is recommended [6, 26, 27]. Gibert et al. isolated *M. capitatus* in a patient with a history of liver transplant transplantation and reported that the patient was treated with voriconazole followed by posaconazole. In the antifungal susceptibility analysis of this isolate using the EUCAST method, the MIC values of voriconazole and posaconazole were lower than those observed in the present study [28]. High MIC values for echinocandins were observed in the isolates from the multicenter prospective study by Fernandez-Ruiz et al. [29]. The study by Esposto et al. also found high MIC values for echinocandins for this species [30].

*Magnusiomyces clavatus* is one of the RYs that cause infections in immunosuppressed patients. Voriconazole and amphotericin B are the preferred treatments [31–33]. In the literature, similar to the results of this study, intrinsic resistance to echinocandins and increased resistance to some azoles have been reported for *M. clavatus* [30, 34, 35]. In vitro resistance to fluconazole and micafungin was reported in the study by Kaplan et al. [33].

Some cases of infection with *M. guilliermondii* while receiving echinocandins have been reported in the literature. It is important to keep in mind that even if the MIC value for echinocandins is low in vitro, clinically there may be reduced susceptibility to these antifungals [36]. In the study by Schmalreck et al., this species exhibited a reduced susceptibility to anidulafungin and caspofungin, in addition to azoles [11]. The study by Stavrou et al. it was reported that amphotericin B was the most effective among 27 isolates whose antifungal susceptibilities were analysed using the EUCAST method, voriconazole was the most effective among triazoles and high MIC values were observed for echinocandins [37]. According to the classification of Astvad et al., all isolates in the present study were susceptible to amphotericin B, three isolates were resistant to anidulafungin, all isolates were resistant to voriconazole, while all isolates were in the intermediate susceptibility category for fluconazole [18]. Pfaller et al. showed that this species has reduced susceptibility to fluconazole, based on isolates collected from 127 medical centers [38].

*Wickerhamomyces anomalus* is an environmental yeast found in plants, soil and other organic matter and has been associated with infections in immunosuppressed patients and outbreaks in healthcare settings [7, 39]. Stavrou et al. found that the majority of *W. anomalus* isolates had high MIC values for fluconazole, while amphotericin B was the most effective agent; the study used the EUCAST method for antifungal susceptibility testing and the results were similar to our results [37]. However, in the study by Chitasombat et al., *W. anomalus* strain were found to be susceptible to azoles, caspofungin and amphotericin B [40]. In the study by Diekema et al., the isolates were reported to have good activity against amphotericin B, fluconazole, voriconazole and echinocandins, with fluconazole showing modest activity [22]. In the study conducted by Lin et al., the identified strains showed good antifungal activity with fluconazole, voriconazole, and micafungin [41].

In the research conducted by Diekema et al. it was observed that the isolates of *C. dubliniensis* were susceptible to triazoles, echinocandins, and amphotericin B [22]. Whereas in a study by Schmalreck et al., there was found to be a reduced susceptibility of *C. dubliniensis* to anidulafungin and micafungin [11]. In the present study, one *C. dubliniensis* isolate demonstrated resistance to anidulafungin and micafungin, while exhibiting susceptibility to other antifungals in a similar manner. The remaining two isolates demonstrated susceptibility to all antifungals according to EUCAST breakpoints. Jean et al. reported that all 107 *C. dubliniensis* isolates were susceptible to amphotericin B, 99.1% were susceptible to fluconazole and voriconazole, and 97.2% were susceptible to posaconazole [42].

The study by Guitard et al. revealed that *P. inconspicua* isolates exhibited resistance to fluconazole, yet demonstrated susceptibility to amphotericin B and echinocandins [43]. Similarly, according to the classification proposed by Astvad et al. in the present study, both isolates exhibited susceptibility to amphotericin B and anidulafungin, while demonstrating resistance to fluconazole and voriconazole [18]. The study by Perez-Hansen et al. also mentioned the increased azole resistance [12]. The study conducted by Kucukoglu et al. observed high MIC values for fluconazole in 30 isolates of this species [13]. A decrease in susceptibility to fluconazole, voriconazole, posaconazole and amphotericin B was also observed in the isolates that were evaluated in the study by Pfaller et al. [21].

*Wickerhamiella pararugosa* is another RY and there are limited number of studies in the literature. Nasri et al. reported that the isolate detected in the blood of a patient with hematological malignancy had a high MIC value for fluconazole and clinical response was obtained with caspofungin treatment [44]. Murata et al. reported that *W. pararugosa* detected in the blood had a high MIC value for fluconazole and was successfully treated with micafungin [44]. In the study by Stavrou et al., low MIC values for amphotericin B, and high MIC values for anidulafungin, micafungin, fluconazole and posaconazole were observed for *W. pararugosa* isalates [37]. In the study by Noni et al. conducted with pediatric patients, it was found that one *W. pararugosa* isolate had a high MIC value for fluconazole but was susceptible to amphotericin B, voriconazole, posaconazole, caspofungin, anidulafungin and micafungin [14]. Interpretation of the MIC values of the isolate in this study according to the classification of Astvad et al. reveals indications of amphotericin B sensitivity and anidulafungin resistance [18].

In the study by Falces-Romero et al., eight *R. mucilaginosa* isolates were identified, and the MIC values for all isolates were found to be high for echinocandins and azoles, and low for amphotericin B [45]. Similar results were detected in this study consistent with the literature. Fernandez-Ruiz et al. demonstrated that strains of *R. mucilaginosa* were resistant to echinocandins and azoles, but susceptible to amphotericin B [29]. Spiliopoulou et al. reported successful treatment with catheter removal and amphotericin B despite high MIC values for amphotericin B in *R. mucilaginosa* isolates [46]. In the study by Noni et al., *R. mucilaginosa* isolate was found to be susceptible to amphotericin B and isavuconazole [14].

*Saccharomyces cerevisiae* is a species commonly used in the food industry and can cause fungemia in immunosuppressed patients, either due to probiotic use or the presence of invasive catheters. If the individual is on probiotics, discontinuation is recommended [47]. In the multicenter study by Schmalreck et al., *S. cerevisiae* isolates demonstrated susceptibility to echinocandins, while exhibited decreased susceptibility to azoles [11]. In the study by Chitasombat et al., *S. cerevisiae* isolates evaluated were found to be susceptible to caspofungin, posaconazole and voriconazole [40]. The isolates in the study by Noni et al. were reported to be susceptible to amphotericin B, echinocandins, voriconazole, and isavuconazole, while high MIC values were observed for fluconazole [14]. In the study by Yan et al., 75 isolates from different body sites were evaluated and it was shown that antifungal susceptibility may vary depending on the anatomical site of isolation [48]. The *S. cerevisiae* isolate in the present study does not appear to be sensitive to fluconazole, voriconazole and anidulafungin according to the classification proposed by Astvad et al. [18].

*Cyberlindnera jadinii* is a species that is frequently utilised within the food industry and is rarely identified as a causative agent of human infections [49]. Studies involving this species are limited in the literature, and case reports have not demonstrated any notable antifungal resistance in vitro [50, 51]. In the case series of neonates and children reported by Singh et al., the isolated strains were found to be susceptible to fluconazole, voriconazole, and amphotericin B [52]. No antifungal resistance was reported in the isolates obtained from the three cases described by Sreelekshmi et al. [53]. Both isolates in the present study were similarly susceptible to amphotericin B, anidulafungin and fluconazole, and moderately susceptible to voriconazole according to the classification of Astvad et al. [18].

Diekema et al. reported that 10 out of 16 *Y. lipolytica* isolates showed reduced susceptibility to amphotericin B. Fluconazole and posaconazole MICs were found to be high, whereas voriconazole and echinocandins showed effective activity against this species [22]. Stavrou et al. reported that *Y. lipolytica* isolates exhibited high MIC values for fluconazole and posaconazole, which are consistent with the findings of the present study. In addition, most isolates were detected to be susceptible to amphotericin B and voriconazole, but resistant to anidulafungin and micafungin [37]. Isolates in the Pfaller et al. study showed reduced susceptibility to fluconazole, voriconazole and posaconazole [21]. The study by Noni et al. reported that *Y. lipolytica* had low MIC values for amphotericin B, echinocandins and azole antifungals other than fluconazole [14].

*Trichosporon asahii* is a species commonly encountered in the urinary system but can also cause invasive disease especially in immunocompromised patients [54]. Invitro antifungal susceptibility studies have shown resistance to echinocandins, while azole groups are generally found to be susceptible [14]. The isolate in the present study can be considered moderately susceptible to fluconazole according to the classification of Astvad et al. [18]. High MIC values for amphotericin B and echinocandins were reported in the isolates from the Fernandez-Ruiz et al. study [29]. Kuo et al. reported that 84 *T. asahii* isolates evaluated in their study showed elevated MIC values for echinocandins. Furthermore, 70.2% of the isolates demonstrated elevated MIC values for fluconazole, while voriconazole and posaconazole were found to be effective [55]. In a multicenter study by Guo et al. in China, the antifungal susceptibility analysis of 108 *T. asahii* species revealed that all isolates demonstrated resistance to echinocandins, with 25% exhibiting elevated MIC values for fluconazole [56].

During the period covered by this study, *C. auris* was considered a RY species, and its presence in our hospital was similarly rare. This rarity was a factor in the decision to include it in our study. However, *C. auris* has since emerged as a significant nosocomial pathogen capable of causing outbreaks in healthcare settings worldwide [15]. In the study by Koleri et al., 36 *C. auris* fungemia were examined and it was found that over 90% of the isolates were resistant to fluconazole and 85% to amphotericin B and 100% were susceptible to echinocandins. Mortality was reported to be 46.1% [15]. In the meta-analysis by Chen et al., over 4000 *C. auris* were evaluated, and it was found that there was high resistance to fluconazole, moderate resistance to amphotericin B and caspofungin, and high sensitivity to anidulafungin and micafungin [16]. The susceptibility results of the isolate in the present study are consistent with these findings. While studies often mention resistance to azoles, especially fluconazole and increasingly to amphotericin B, some studies also report resistance to echinocandins [57, 58].

Factors facilitating fungal infections, such as malignancies, surgical history, ICU admission, neutropenia, TPN and the presence of central venous catheters, have been frequently investigated [29, 59]. The patients in this study were found to have the majority of these risk factors. Kutlu et al. found a significant correlation between 30-day mortality in candidemia and TPN, high sequential organ failure assessment score, antibiotic exposure, newly developed renal failure and newly developed thrombocytopenia. The study included 425 patients, and the 30-day mortality rate was 56.7%, which is higher than the rate in this study [60]. A multicenter prospective study of RYs by Fernandez-Ruiz et al. reported a 30-day mortality rate of 46.2% [29]. Del Principe et al. in a multicenter study of patients with hematological malignancy infected with *M. clavatus* or *M. capitatus*, 30-day mortality was found to be 43% and septic shock, treatment with corticosteroids for more than 14 days and lack of neutrophil recovery were associated with high mortality [61]. In a multicenter study conducted by Gil et al. in Latin America, RY infections were evaluated and the crude mortality rate was reported as 40.8% [62].

There were some limitations to this study. Firstly, due to the retrospective design of the study, we could not access some clinical information about the patients. There were some deficiencies in recording the data of patients in some clinics, which may be attributed to the impact of the Coronavirus Diseases 2019 pandemic. Secondly, some isolates initially planned for inclusion in the study were excluded due to lack of viability. Hence, the number and variety of isolates examined decreased. Thirdly, due to the single-center nature of the study, a limited number of isolates from a limited number of patients were included. Fourthly the high diversity of RY fungi and the heterogeneity of the patients posed limitations in evaluating the risk factors facilitating the infection. Finally, there was lack of molecular identification methods, which could have further supported species-level identification.

The aim of this study was to investigate patients with invasive RY and to examine the in vitro antifungal susceptibility analyses of the isolates. We found varying results regarding antifungal susceptibility analyses among different species. Most strains showed high MIC values for fluconazole and relatively low MIC values for amphotericin B.

Thirty-day mortality was significantly associated with age 65 years or older, neutropenia, and ICU admission, and fourteen-day mortality was also significantly associated with age 65 years or older and ICU admission. Studies of RYs are often limited to small numbers of patients or presented in the form of case reports. However, this study examined slightly larger number of patients.

## Conclusions

In conclusion, this study provides different ranges of antifungal susceptibility results for different RY species. This should be taken into consideration when initiating appropriate antifungal treatment. The variability of RY encountered in various centers and differences in antifungal susceptibility profiles warrant further research on this topic, both regionally and globally.

## Electronic supplementary material

Below is the link to the electronic supplementary material.

Supplementary Material 1

## Data Availability

All data generated or analysed during this study are included in this published article.

## Abbreviations

CI: Confidence interval
ECOFF: Epidemiological cut-off
EUCAST: European Committee on Antimicrobial Susceptibility Testing
HR: Hazard ratio
ICU: Intensive care unit
IFI: Invasive fungal infection
IQR: Interquartile range
MALDITOF MS: Matrix-assisted laser desorption ionization time of flight mass spectrometry
MIC: Minimum inhibitory concentration
RY: Rare yeast
TPN: Total parenteral nutrition

## References

[1] Miceli MH, Diaz JA, Lee SA. Emerging opportunistic yeast infections. Lancet Infect Dis. 2011;11(2):142–51. 21272794 10.1016/S1473-3099(10)70218-8

[2] McCarty TP, White CM, Pappas PG. Candidemia and invasive candidiasis. Infect Dis Clin N Am. 2021;35(2):389–413. 10.1016/j.idc.2021.03.007 34016283

[3] Ghasemi R, Lotfali E, Rezaei K, Madinehzad SA, Tafti MF, Aliabadi N, et al. Meyerozyma guilliermondii species complex: review of current epidemiology, antifungal resistance, and mechanisms. Braz J Microbiol. 2022;53(4):1761–79. 36306113 10.1007/s42770-022-00813-2 PMC9679122

[4] Sendid B, Lacroix C, Bougnoux ME. Is Candida Kefyr an emerging pathogen in patients with oncohematological diseases? Clin Infect Dis. 2006;43(5):666–7. 16886166 10.1086/506573

[5] Apsemidou A, Fuller MA, Idelevich EA, Kurzai O, Tragiannidis A, Groll AH. Candida lusitaniae breakthrough fungemia in an Immuno-Compromised adolescent: case report and review of the literature. J fungi (Basel Switzerland). 2020;6(4). 10.3390/jof6040380 PMC7766689 33371186

[6] Martino R, Salavert M, Parody R, Tomas JF, de la Camara R, Vazquez L, et al. Blastoschizomyces capitatus infection in patients with leukemia: report of 26 cases. Clin Infect Dis. 2004;38(3):335–41. 14727202 10.1086/380643

[7] Pasqualotto AC, Sukiennik TC, Severo LC, de Amorim CS, Colombo AL. An outbreak of Pichia anomala fungemia in a Brazilian pediatric intensive care unit. Infect Control Hosp Epidemiol. 2005;26(6):553–8. 16018431 10.1086/502583

[8] Sprute R, Cornely OA, Chen SC, Seidel D, Schuetz AN, Zhang SX. All you need to know and more about the diagnosis and management of rare yeast infections. mBio. 2021;12(4):e0159421. 34425700 10.1128/mBio.01594-21 PMC8406212

[9] Yang Y, Guo F, Kang Y, Zang B, Cui W, Qin B, et al. Epidemiology, clinical characteristics, and risk factors for mortality of early- and late-onset invasive candidiasis in intensive care units in China. Med (Baltim). 2017;96(42):e7830. 10.1097/MD.0000000000007830 PMC5662350 29049184

[10] Xiao M, Chen SC, Kong F, Fan X, Cheng JW, Hou X, et al. Five-year China hospital invasive fungal surveillance net (CHIF-NET) study of invasive fungal infections caused by noncandidal yeasts: species distribution and Azole susceptibility. Infect Drug Resist. 2018;11:1659–67. 30349323 10.2147/IDR.S173805 PMC6183553

[11] Schmalreck AF, Willinger B, Haase G, Blum G, Lass-Flörl C, Fegeler W, et al. Species and susceptibility distribution of 1062 clinical yeast isolates to Azoles, echinocandins, Flucytosine and amphotericin B from a multi-centre study. Mycoses. 2012;55(3):e124–37. 22233267 10.1111/j.1439-0507.2011.02165.x

[12] Perez-Hansen A, Lass-Florl C, Lackner M, Rare Yeast Study G. Antifungal susceptibility profiles of rare ascomycetous yeasts. J Antimicrob Chemother. 2019;74(9):2649–56. 31203366 10.1093/jac/dkz231

[13] Kucukoglu O, Sariguzel FM, Koc AN, Parkan OM. Molecular epidemiology, virulence factors, and antifungal susceptibility of Candida inconspicua strains isolated from clinical samples in Turkey. Diagn Microbiol Infect Dis. 2023;106(1):115915. 36947944 10.1016/j.diagmicrobio.2023.115915

[14] Noni M, Stathi A, Velegraki A, Malamati M, Kalampaliki A, Zachariadou L et al. Rare invasive yeast infections in Greek neonates and children, a retrospective 12-Year study. J fungi (Basel Switzerland). 2020;6(4). 10.3390/jof6040194 PMC7711555 32998455

[15] Koleri J, Petkar HM, Rahman SASHA, Rahman SAMA. Candida auris blood stream infection- a descriptive study from Qatar. BMC Infect Dis. 2023;23(1):513. 37544995 10.1186/s12879-023-08477-5 PMC10405369

[16] Chen J, Tian S, Han X, Chu Y, Wang Q, Zhou B, et al. Is the Superbug fungus really so scary? A systematic review and meta-analysis of global epidemiology and mortality of Candida auris. BMC Infect Dis. 2020;20(1):827. 33176724 10.1186/s12879-020-05543-0 PMC7656719

[17] Method for the Determination of Broth Dilution Minimum Inhibitory Concentrations of Antifungal Agents for Yeasts, Version 7.3.2. [Internet]. 2020. Available from: https://www.eucast.org/fileadmin/src/media/PDFs/EUCAST_files/AFST/Files/EUCAST_E_Def_7.3.2_Yeast_testing_definitive_revised_2020.pdf

[18] Astvad KMT, Arikan-Akdagli S, Arendrup MC. A pragmatic approach to susceptibility classification of yeasts without EUCAST clinical breakpoints. J Fungi (Basel). 2022;8(2). 10.3390/jof8020141 PMC8877802 35205895

[19] Alp S, Gulmez D, Kardas RC, Karahan G, Tas Z, Gursoy G, et al. Expect the unexpected: fungemia caused by uncommon Candida species in a Turkish university hospital. Eur J Clin Microbiol Infect Dis. 2021;40(7):1539–45. 33495941 10.1007/s10096-020-04147-5

[20] Peyron F, Favel A, Michel-Nguyen A, Gilly M, Regli P, Bolmstrom A. Improved detection of amphotericin B-resistant isolates of Candida lusitaniae by etest. J Clin Microbiol. 2001;39(1):339–42. 11136795 10.1128/JCM.39.1.339-342.2001 PMC87726

[21] Pfaller MA, Diekema DJ, Messer SA, Boyken L, Hollis RJ, Jones RN, et al. In vitro activities of voriconazole, posaconazole, and four licensed systemic antifungal agents against Candida species infrequently isolated from blood. J Clin Microbiol. 2003;41(1):78–83. 12517829 10.1128/JCM.41.1.78-83.2003 PMC149631

[22] Diekema DJ, Messer SA, Boyken LB, Hollis RJ, Kroeger J, Tendolkar S, et al. In vitro activity of seven systemically active antifungal agents against a large global collection of rare Candida species as determined by CLSI broth microdilution methods. J Clin Microbiol. 2009;47(10):3170–7. 19710283 10.1128/JCM.00942-09 PMC2756931

[23] Spiliopoulou A, Kolonitsiou F, Vrioni G, Tsoupra S, Lekkou A, Paliogianni F. Invasive Candida Kefyr infection presenting as pyelonephritis in an ICU hospitalized COVID-19 patient: case report and review of the literature. J Mycol Med. 2022;32(2):101236. 34974339 10.1016/j.mycmed.2021.101236 PMC8694783

[24] Tanabe MB, Patel SA. Blastoschizomyces capitatus pulmonary infections in immunocompetent patients: case report, case series and literature review. Epidemiol Infect. 2018;146(1):58–64. 29198203 10.1017/S0950268817002643 PMC9134742

[25] Mazzocato S, Marchionni E, Fothergill AW, Sutton DA, Staffolani S, Gesuita R, et al. Epidemiology and outcome of systemic infections due to saprochaete capitata: case report and review of the literature. Infection. 2015;43(2):211–5. 25078793 10.1007/s15010-014-0668-3

[26] Cavanna C, Lallitto F, Mangione F, Tamarozzi F, Marone P, Ceriana P. Fungemia due to saprochaete capitata in a non-neutropenic patient hospitalized in an intensive care unit after cardiac surgery. J Med Mycol. 2017;27(2):281–4. 10.1016/j.mycmed.2017.01.014 28302347

[27] Brunetti G, Visconti V, Ghezzi MC, Mantovani S, Ferretti G, Raponi G. Management and treatment of Magnusiomyces capitatus (Geotrichum capitatum) pleural infection in a non-neutropenic patient with posaconazole. A new therapeutic opportunity? New Microbiol. 2016;39(4):307–9. 27284991

[28] Gibert C, Wan M, Arsicot M, Huvelle U, Penhoat T, Koumar Y, et al. Mycotic aneurysm due to Magnusiomyces capitatus complicating a second liver transplant in a colonized patient. Int J Infect Dis. 2025;151:107370. 39710137 10.1016/j.ijid.2024.107370

[29] Fernandez-Ruiz M, Guinea J, Puig-Asensio M, Zaragoza O, Almirante B, Cuenca-Estrella M, et al. Fungemia due to rare opportunistic yeasts: data from a population-based surveillance in Spain. Med Mycol. 2017;55(2):125–36. 27495321 10.1093/mmy/myw055

[30] Esposto MC, Prigitano A, Lo Cascio G, Ossi C, Grancini A, Cavanna C, et al. Yeast-like filamentous fungi: molecular identification and in vitro susceptibility study. Med Mycol. 2019;57(7):909–13. 30521007 10.1093/mmy/myy133

[31] Buchta V, Bolehovská R, Hovorková E, Cornely OA, Seidel D, Žák P. Saprochaete clavata invasive Infections - A new threat to Hematological-Oncological patients. Front Microbiol. 2019;10:2196. 31736883 10.3389/fmicb.2019.02196 PMC6830389

[32] Favre S, Rougeron A, Levoir L, Pérard B, Milpied N, Accoceberry I, et al. Saprochaete clavata invasive infection in a patient with severe aplastic anemia: efficacy of voriconazole and liposomal amphotericin B with adjuvant granulocyte transfusions before neutrophil recovery following allogeneic bone marrow transplantation. Med Mycol Case Rep. 2016;11:21–3. 27069848 10.1016/j.mmcr.2016.03.001 PMC4811851

[33] Kaplan E, Al-Hatmi AMS, Ilkit M, van den Gerrits AHG, Hagen F, Meis JF et al. Molecular diagnostics of arthroconidial yeasts, frequent pulmonary opportunists. J Clin Microbiol. 2018;56(1). 10.1128/JCM.01427-17 PMC5744204 29070656

[34] Noster J, Koeppel MB, Desnos-Olivier M, Aigner M, Bader O, Dichtl K, et al. Bloodstream infections caused by Magnusiomyces capitatus and Magnusiomyces clavatus: epidemiological, clinical, and Microbiological features of two emerging yeast species. Antimicrob Agents Chemother. 2022;66(2):e0183421. 34930027 10.1128/aac.01834-21 PMC8846490

[35] Desnos-Ollivier M, Lortholary O, Bretagne S, Dromer F. Azole susceptibility profiles of more than 9,000 clinical yeast isolates belonging to 40 common and rare species. Antimicrob Agents Chemother. 2021;65(6). 10.1128/AAC.02615-20 PMC8315974 33820766

[36] Kabbara N, Lacroix C, Peffault de Latour R, Socie G, Ghannoum M, Ribaud P, Breakthrough C. Parapsilosis and C. guilliermondii blood stream infections in allogeneic hematopoietic stem cell transplant recipients receiving long-term Caspofungin therapy. Haematologica. 2008;93(4):639–40. 18379015 10.3324/haematol.11149

[37] Stavrou AA, Perez-Hansen A, Lackner M, Lass-Florl C, Boekhout T. Elevated minimum inhibitory concentrations to antifungal drugs prevail in 14 rare species of candidemia-causing Saccharomycotina yeasts. Med Mycol. 2020;58(7):987–95. 32043147 10.1093/mmy/myaa005

[38] Pfaller MA, Diekema DJ, Mendez M, Kibbler C, Erzsebet P, Chang SC, et al. Candida guilliermondii, an opportunistic fungal pathogen with decreased susceptibility to fluconazole: geographic and Temporal trends from the ARTEMIS DISK antifungal surveillance program. J Clin Microbiol. 2006;44(10):3551–6. 17021081 10.1128/JCM.00865-06 PMC1594787

[39] Aragão PA, Oshiro IC, Manrique EI, Gomes CC, Matsuo LL, Leone C, et al. Pichia anomala outbreak in a nursery: exogenous source? Pediatr Infect Dis J. 2001;20(9):843–8. 11734761 10.1097/00006454-200109000-00004

[40] Chitasombat MN, Kofteridis DP, Jiang Y, Tarrand J, Lewis RE, Kontoyiannis DP. Rare opportunistic (non-Candida, non-Cryptococcus) yeast bloodstream infections in patients with cancer. J Infect. 2012;64(1):68–75. 22101079 10.1016/j.jinf.2011.11.002 PMC3855381

[41] Lin HC, Lin HY, Su BH, Ho MW, Ho CM, Lee CY, et al. Reporting an outbreak of Candida pelliculosa fungemia in a neonatal intensive care unit. J Microbiol Immunol Infect. 2013;46(6):456–62. 23050985 10.1016/j.jmii.2012.07.013

[42] Jean SS, Yang HJ, Hsieh PC, Huang YT, Ko WC, Hsueh PR. In vitro susceptibilities of worldwide isolates of intrapulmonary Aspergillus species and important Candida species in sterile body sites against important antifungals: data from the antimicrobial testing leadership and surveillance program, 2017–2020. Microbiol Spectr. 2022;10(6):e0296522. 36314941 10.1128/spectrum.02965-22 PMC9769544

[43] Guitard J, Angoulvant A, Letscher-Bru V, L’Ollivier C, Cornet M, Dalle F, et al. Invasive infections due to Candida norvegensis and Candida inconspicua: report of 12 cases and review of the literature. Med Mycol. 2013;51(8):795–9. 23855412 10.3109/13693786.2013.807444

[44] Murata S, Mimura K, Kawamura T, Saito H, Ohno H, Tsujii E, et al. Bloodstream infection caused by Wickerhamiella Pararugosa in a patient with intestinal obstruction: A case report. J Infect Chemother. 2024;30(9):942–5. 38369124 10.1016/j.jiac.2024.02.014

[45] Falces-Romero I, Cendejas-Bueno E, Romero-Gomez MP, Garcia-Rodriguez J. Isolation of Rhodotorula mucilaginosa from blood cultures in a tertiary care hospital. Mycoses. 2018;61(1):35–9. 28922488 10.1111/myc.12703

[46] Spiliopoulou A, Lekkou A, Vrioni G, Leonidou L, Cogliati M, Christofidou M, et al. Fungemia due to rare non-Candida yeasts between 2018 and 2021 in a Greek tertiary care university hospital. J Med Mycol. 2023;33(3):101386. 10.1016/j.mycmed.2023.101386 37031651

[47] Roy U, Jessani LG, Rudramurthy SM, Gopalakrishnan R, Dutta S, Chakravarty C, et al. Seven cases of Saccharomyces fungaemia related to use of probiotics. Mycoses. 2017;60(6):375–80. 28133894 10.1111/myc.12604

[48] Yan Z, Fu Y, Tan X, Xu L, Ling J, Liu X, et al. Isolate distribution and antifungal susceptibility of Saccharomyces cerevisiae in the National regional medical center of Southwest China for women and children during 2018–2023. BMC Microbiol. 2024;24(1):345. 39271978 10.1186/s12866-024-03506-y PMC11401246

[49] Holzschu DL, Chandler FW, Ajello L, Ahearn DG. Evaluation of industrial yeasts for pathogenicity. Sabouraudia. 1979;17(1):71–8. 441902 10.1080/00362177985380091

[50] Scoppettuolo G, Donato C, De Carolis E, Vella A, Vaccaro L, La Greca A, et al. Candida utilis catheter-related bloodstream infection. Med Mycol Case Rep. 2014;6:70–2. 25473600 10.1016/j.mmcr.2014.10.003 PMC4246400

[51] Gaisne R, Jeddi F, Morio F, Le Clerc QC, Hourmant M, Blancho G, et al. Candida utilis fungaemia following endoscopic intervention on ureteral stent in a kidney transplant recipient: case report and a review of the literature. Mycoses. 2018;61(8):594–9. 29575315 10.1111/myc.12766

[52] Singh DP, Verma RK, Yadav RK, Anant K, Das A. Candida utilis as an emerging rare cause of septicemia among neonates and children in Western Uttar Pradesh: A case series. Curr Med Mycol. 2024;10:e2024.345184.1529. 10.22034/cmm.2024.345218.1529 PMC11688595 39744335

[53] Sreelekshmi TS, Ninan MM, Premanand A, Chacko A, Sahni RD, Michael JS. Candida utilis: a rare cause of septicemia in children. Access Microbiol. 2021;3(10):000281. 34816095 10.1099/acmi.0.000281 PMC8604182

[54] Francisco EC, de Almeida Junior JN, de Queiroz Telles F, Aquino VR, Mendes AVA, de Andrade Barberino MGM, et al. Species distribution and antifungal susceptibility of 358 trichosporon clinical isolates collected in 24 medical centres. Clin Microbiol Infect. 2019;25(7):909.e1-.e5. 10.1016/j.cmi.2019.03.026 30991116

[55] Kuo SH, Lu PL, Chen YC, Ho MW, Lee CH, Chou CH, et al. The epidemiology, genotypes, antifungal susceptibility of trichosporon species, and the impact of voriconazole on trichosporon fungemia patients. J Formos Med Assoc. 2021;120(9):1686–94. 33358563 10.1016/j.jfma.2020.12.007

[56] Guo LN, Yu SY, Hsueh PR, Al-Hatmi AMS, Meis JF, Hagen F et al. Invasive infections due to Trichosporon: species distribution, genotyping, and antifungal susceptibilities from a multicenter study in China. J Clin Microbiol. 2019;57(2). 10.1128/JCM.01505-18 PMC6355529 30463892

[57] de St Maurice A, Parti U, Anikst VE, Harper T, Mirasol R, Dayo AJ, et al. Clinical, Microbiological, and genomic characteristics of clade-III Candida auris colonization and infection in Southern California, 2019–2022. Infect Control Hosp Epidemiol. 2023;44(7):1093–101. 36052507 10.1017/ice.2022.204 PMC10369217

[58] Biran R, Cohen R, Finn T, Brosh-Nissimov T, Rahav G, Yahav D, et al. Nationwide outbreak of Candida auris infections driven by COVID-19 hospitalizations, Israel, 2021–2022. Emerg Infect Dis. 2023;29(7):1297–301. 37347492 10.3201/eid2907.221888 PMC10310389

[59] Kontoyiannis DP, Torres HA, Chagua M, Hachem R, Tarrand JJ, Bodey GP, et al. Trichosporonosis in a tertiary care cancer center: risk factors, changing spectrum and determinants of outcome. Scand J Infect Dis. 2004;36(8):564–9. 15370667 10.1080/00365540410017563

[60] Kutlu M, Sayin-Kutlu S, Alp-Cavus S, Ozturk SB, Tasbakan M, Ozhak B, et al. Mortality-associated factors of candidemia: a multi-center prospective cohort in Turkey. Eur J Clin Microbiol Infect Dis. 2022;41(4):597–607. 35083558 10.1007/s10096-021-04394-0

[61] Del Principe MI, Seidel D, Criscuolo M, Dargenio M, Rácil Z, Piedimonte M, et al. Clinical features and prognostic factors of Magnusiomyces (Saprochaete) infections in haematology. A multicentre study of SEIFEM/Fungiscope. Mycoses. 2023;66(1):35–46. 36064299 10.1111/myc.13524

[62] Gil Ó, Hernández-Pabón JC, Tabares B, Lugo-Sánchez C, Firacative C. Rare yeasts in Latin America: uncommon yet meaningful. J Fungi (Basel). 2023;9(7). 10.3390/jof9070747 PMC10381163 37504735

