# Supplementary material for: Epidemiology and antifungal susceptibilities of rare yeast infections in a tertiary care center
Source: BMC Microbiol. 2025 Jul 2;25:385. doi: 10.1186/s12866-025-04016-1 (PMC12219992; doi:10.1186/s12866-025-04016-1)
Supplement: Supplementary file 1 — Supplementary Material 1 [file 12866_2025_4016_MOESM1_ESM.docx]

**Supplementary table 1. Detailed patient characteristics, and antifungal susceptibilities of rare yeasts**

| **Patients’ clinical information** | | | | **Antifungal susceptibility results, MIC values (mg/L)** | | | | | | | | |
| --- | --- | --- | --- | --- | --- | --- | --- | --- | --- | --- | --- | --- |
| **A/G^a^** | **Clinical features** | **Treatment** | **Outcome** | **Species** | **AMB** | **FLC** | **VRC** | **POS** | **ISA** | **AFG** | **MFG** | **CAS** |
| 74/M | Cerebrovascular accident, ICU follow-up | CAS | Death | *Clavispora lusitaniae* | 0.031 | 0.125 | <0.008 | 0.0625 | <0.008 | 0.25 | 0.0625 | 0.5 |
| 19/M | Burn injuries, ICU follow-up | AFG | Death | *Clavispora lusitaniae* | 0.25 | 4 | 0.25 | 0.25 | 0.125 | 4 | 0.5 | 1 |
| 51/F | Breast cancer | Died before diagnosis | Death | *Clavispora lusitaniae* | 0.25 | 0.25 | <0.008 | 0.015 | <0.008 | 0.25 | 0.125 | 0.25 |
| 3/M | Sandhoff syndrome, ICU follow-up | FLC | Alive | *Clavispora lusitaniae* | 0.0625 | 0.25 | 0.008 | <0.008 | <0.008 | 0.031 | 0.0625 | 0.5 |
| 4/F | West syndrome, ICU follow-up | FLC | Death | *Clavispora lusitaniae* | 0.5 | 0.25 | 0.008 | 0.015 | <0.008 | 0.0625 | <0.008 | 0.25 |
| 3/M | MHC Class 2 deficiency, autologous stem cell transplant, ICU follow-up | Died before diagnosis | Death | *Clavispora lusitaniae* | 0.25 | <0.125 | <0.008 | 0.008 | <0.008 | 0.125 | 0.0625 | 0.25 |
| 59/M | Bladder cancer, HT, DM, coronary artery disease | FLC | Alive | *Clavispora lusitaniae* | 0.125 | 0.25 | <0.008 | 0.0625 | <0.008 | 0.015 | 0.0625 | 0.25 |
| 65/M | Hepatocellular carcinoma | Died before diagnosis | Death | *Clavispora lusitaniae* | 0.25 | 0.5 | 0.008 | 0.0625 | <0.008 | 0.125 | 0.0625 | 0.125 |
| 80/M | Colon cancer | NA | Alive | *Clavispora lusitaniae* | 0.125 | 0.25 | <0.008 | 0.015 | <0.008 | 0.031 | 0.031 | 0.5 |
| 43/M | Burn injuries, ICU follow-up | CAS | Alive | *Clavispora lusitaniae* | 0.125 | 1 | 0.015 | 0.008 | <0.008 | 0.125 | 0.031 | 0.25 |
| 77/M | Lung tuberculosis, CKD, cerebrovascular accident | FLC | Alive | *Clavispora lusitaniae* | 0.031 | 0.5 | 0.015 | 0.031 | <0.008 | 0.125 | 0.0625 | 0.25 |
| 97/F | HT, heart failure, ICU follow-up | FLC | Death | *Clavispora lusitaniae* | 0.25 | 0.5 | <0.008 | 0.125 | <0.008 | 0.0625 | 0.0625 | 0.25 |
| 1/F | Esophageal atresia, ICU follow-up | FLC | Alive | *Clavispora lusitaniae* | 0.25 | 0.5 | 0.008 | 0.0625 | <0.008 | 0.125 | 0.125 | 0.25 |
| 56/F | Cerebrovascular accident | NA | Alive | *Clavispora lusitaniae* | 0.25 | 0.125 | <0.008 | <0.008 | <0.008 | 0.25 | 0.031 | 0.5 |
| 2/F^b^ | SCID, ICU follow-up | CAS | Alive | *Kluyveromyces marxianus* | 0.25 | 4 | 0.0625 | 0.5 | <0.008 | 0.25 | 0.125 | 0.0625 |
| 1/M | Atypical HUS, pontocerebellar hypoplasia, ICU follow-up | MFG | Alive | *Kluyveromyces marxianus* | 0.0625 | 0.5 | 0.015 | 0.015 | <0.008 | <0.008 | <0.008 | 0.031 |
| 74/M | Central nervous system tumor, ICU follow-up | Refused treatment | Death | *Kluyveromyces marxianus* | 0.25 | 0.5 | 0.015 | 0.008 | <0.008 | 0.25 | 0.031 | 0.0625 |
| 56/M | DLBCL, ICU follow-up | AMB | Death | *Kluyveromyces marxianus* | 0.25 | 32 | 0.25 | 0.5 | 0.125 | 0.125 | 0.125 | 0.25 |
| 75/M | AML | VRC | Death | *Kluyveromyces marxianus* | 0.5 | 0.5 | 0.5 | 0.125 | <0.008 | 0.25 | 0.031 | 0.031 |
| 20/M | T-cell ALL | FLC | Alive | *Kluyveromyces marxianus* | 0.5 | 1 | 0.125 | 0.25 | <0.008 | 0.25 | 0.0625 | 0.031 |
| 60/M | Liver transplantation, coronary artery disease, hypothyroidism, ICU follow-up | AFG | Alive | *Kluyveromyces marxianus* ^c^ | 0.125 | 0.5 | <0.008 | 0.0625 | <0.008 | 0.125 | 0.031 | 0.0625 |
| 59/M | Central nervous system tumor, gastric perforation, ICU follow-up | Died before diagnosis | Death | *Kluyveromyces marxianus* | 0.5 | 0.5 | <0.008 | <0.008 | <0.008 | 0.125 | 0.015 | 0.031 |
| 2/M | Neuroblastoma | FLC | Alive | *Kluyveromyces marxianus* | 0.5 | 16 | 2 | 0.5 | 0.125 | 0.031 | 0.125 | 0.5 |
| 39/F | Cervical cancer | FLC | Alive | *Kluyveromyces marxianus* | 0.25 | 1 | 0.015 | 0.125 | <0.008 | 0.25 | 0.125 | 0.0625 |
| 44/F | Renal transplantation, DM | FLC | Alive | *Kluyveromyces marxianus* | 0.25 | 0.25 | <0.008 | 0.008 | <0.008 | 0.125 | 0.031 | 0.125 |
| 5/M | Neuroblastoma, autologous stem cell transplantation | CAS + VRC | Alive | *Kluyveromyces marxianus* | 0.5 | 32 | 0.25 | 0.5 | 0.125 | 0.125 | 0.125 | 0.5 |
| 15/M | Burkitt lymphoma, ICU follow-up | AMB + VRC | Death | *Magnusiomyces capitatus* | 0.5 | 64 | >4 | 4 | 4 | >4 | >4 | >4 |
| 16/M | AML | AMB | Alive | *Magnusiomyces capitatus* | 0.5 | >64 | >4 | 4 | >4 | >4 | >4 | >4 |
| 7/M | Cerebral palsy, ICU follow-up | MFG | Alive | *Magnusiomyces capitatus* | 0.5 | >64 | >4 | 4 | >4 | >4 | >4 | > |
| 66/M | AML | VRC | Alive | *Magnusiomyces capitatus* | 1 | 64 | 4 | >4 | 2 | >4 | >4 | >4 |
| 55/M | Heart transplantation | NA | Alive | *Magnusiomyces capitatus* | 1 | 32 | 2 | >4 | >4 | >4 | >4 | >4 |
| 8/M | T- cell ALL | AMB | Alive | *Wickerhamomyces anomalus* | 0.25 | 4 | 0.125 | 0.25 | <0.008 | 0.008 | 0.008 | 0.25 |
| 43/F | Larynx cancer | FLC | Alive | *Wickerhamomyces anomalus* | 0.0625 | 4 | 0.125 | 0.0625 | 0.008 | 0.008 | <0.008 | 0.125 |
| 6/F | Renal transplantation | CAS | Alive | *Wickerhamomyces anomalus* | 0.125 | 4 | 0.125 | 2 | 0.008 | 0.031 | <0.008 | 0.125 |
| 68/F | Ovarian cancer, HT | NA | Alive | *Wickerhamomyces anomalus* | 0.015 | 4 | 0.25 | 1 | 0.031 | 0.0625 | <0.008 | 0.125 |
| 9/M | B-cell ALL, ICU follow-up, prior antifungal use for IPA (AMB and VRC) | AMB + VRC + CAS | Death | *Meyerozyma guilliermondii* | 0.0625 | 8 | 0.5 | 0.5 | 0.25 | 4 | 0.25 | 0.5 |
| 1/M | AML, allogeneic stem cell transplantation | AMB + CAS | Alive | *Meyerozyma guilliermondii* | 0.25 | 4 | 0.25 | 0.5 | 0.25 | 2 | 0.5 | 1 |
| 9/M^b^ | AML, allogeneic stem cell transplantation | AMB + CAS | Alive | *Meyerozyma guilliermondii* | 0.5 | 8 | 0.25 | 0.5 | 0.5 | 2 | 0.25 | 0.5 |
| 2/F^b^ | SCID, ICU follow-up | CAS | Alive | *Meyerozyma guilliermondii* | 0.125 | 8 | 0.25 | 0.5 | 0.5 | 0.125 | 0.25 | 0.5 |
| 71/M | Lung cancer, CKD, ICU follow-up | Died before diagnosis | Death | *Magnusiomyces clavatus* | 0.25 | >4 | >4 | 4 | >4 | >4 | >4 | >4 |
| 57/M | Renal transplantation, ICU follow-up, prior antifungal use for mucormycosis | AMB | Alive | *Magnusiomyces clavatus* | 0.25 | >64 | >4 | >4 | >4 | >4 | 0.25 | >4 |
| 51/M | B-cell ALL, HT, CKD, ICU follow-up, prior antifungal use for IPA (VRC followed by AMB) | Died before diagnosis | Death | *Magnusiomyces clavatus* | 0.25 | 2 | 0.015 | 1 | 0.008 | >4 | >4 | >4 |
| 18/M | Short bowel syndrome | FLC | Alive | *Candida dubliniensis* | 0.125 | 0.25 | 0.008 | 0.015 | <0.008 | 0.008 | 0.008 | 0.0625 |
| 51/M | Mesenteric ischemia | FLC | Alive | *Candida dubliniensis* | 0.25 | 0.5 | <0.008 | 0.031 | <0.008 | 0.008 | 0.008 | 0.0625 |
| 68/F | Parkinson, HT, ICU follow-up | FLC | Alive | *Candida dubliniensis* | 0.125 | 0.5 | 0.015 | 0.015 | <0.008 | 4 | 1 | 1 |
| 18/F | Down syndrome, lymphoblastic leukemia | CAS | Alive | *Pichia inconspicua* | 0.125 | 32 | >4 | 1 | 0.125 | <0.008 | 0.031 | 0.5 |
| 9/M | Central nervous system tumor | CAS | Alive | *Pichia inconspicua* | 0.25 | >64 | 0.5 | 0.25 | 0.25 | 0.0625 | 0.031 | 0.015 |
| 72/F | DLBCL, ICU follow-up | Died before diagnosis | Death | *Cyberlindnera jadinii* | 0.125 | 1 | 0.0625 | <0.008 | 0.008 | 0.015 | <0.008 | 1 |
| 39/M | Workplace accident (multi-trauma), ICU follow-up | FLC | Alive | *Cyberlindnera jadinii* | 0.0625 | 2 | 0.125 | 0.25 | 0.008 | 0.015 | <0.008 | 0.031 |
| 43/M | Pancreatic cancer | Refused treatment | Death | *Rhodotorula mucilaginosa* | 1 | 64 | >4 | >4 | >4 | >4 | >4 | >4 |
| 3/M | MSUD, liver transplantation, ICU follow-up | AMB followed by VRC | Alive | *Rhodotorula mucilaginosa* | 0.5 | >64 | 1 | 0.5 | 1 | >4 | >4 | >4 |
| 64/M | AML | CAS | Alive | *Saccharomyces cerevisiae* | 1 | >64 | >4 | >4 | >4 | >4 | 0.0625 | 1 |
| 88/M | HT, DM, ICU follow-up | MFG | Death | *Yarrowia lipolytica* | 0.5 | 8 | >4 | >4 | >4 | 0.5 | 1 | 1 |
| 27/M | DLBCL, ICU follow-up | MFG | Death | *Candida auris* | 0.5 | 64 | 0.25 | 0.125 | <0.008 | 0.5 | 0.0625 | 0.125 |
| 35/M | Neurobrucellosis | NA | Alive | *Trichosporon asahii* | 0.031 | 2 | 0.031 | 0.5 | 0.008 | >4 | >4 | >4 |
| 9/M^b^ | AML, allogeneic stem cell transplantation | AMB + CAS | Alive | *Wickerhamiella pararugosa* | 0.125 | 1 | 0.25 | 0.25 | 0.25 | 1 | 2 | 0.5 |

^a^ Age (year)/Gender

^b^ Patients who have been hospitalized for more than six months and are diagnosed with more than one invasive fungal infection.

^c^ Pleural culture

ALL, acute lymphoblastic leukemia; AML, acute myeloid leukemia; AMB, amphotericin B; AFG, anidulafungin; CAS, caspofungin; CKD, chronic, kidney disease; DLBCL, diffuse large B-cell lymphoma; DM, diabetes mellitus; M, male; FLC, fluconazole; HT, hypertension; HUS, hemolytic uremic syndrome; IPA, invasive pulmonary aspergillosis; ISA, isavuconazole; F, female; MDS, myelodysplastic syndrome; MFG, micafungin; MSUD, maple syrup urine disease; POS, posaconazole; SCID, severe combined immunodeficiency disease; VRC, voriconazole.
